# Impact of Electrode Surface Morphology in ZnO-Based Resistive Random Access Memory Fabricated Using the Cu Chemical Displacement Technique

**DOI:** 10.3390/ma11020265

**Published:** 2018-02-09

**Authors:** Chi-Chang Wu, Hsin-Chiang You, Yu-Hsien Lin, Chia-Jung Yang, Yu-Ping Hsiao, Tun-Po Liao, Wen-Luh Yang

**Affiliations:** 1 Department of Electronic Engineering, Feng Chia University, Taichung 40724, Taiwan; cchangwu@fcu.edu.tw (C.-C.W.); sky711363@gmail.com (C.-J.Y.); heavenxaw11@yahoo.com.tw (Y.-P.H.); bb830218@gmail.com (T.-P.L.); 2 Department of Electronic Engineering, National Chin-Yi University of Technology, Taichung 41170, Taiwan; hcyou@ncut.edu.tw; 3 Department of Electronic Engineering, National United University, Miaoli 36003, Taiwan; yhlin@nuu.edu.tw

**Keywords:** ECM, resistive memory, ReRAM, chemical displacement, Cu-CDT

## Abstract

Electrochemical-metallization-type resistive random access memories (ReRAMs) show promising performance as next-generation nonvolatile memory. In this paper, the Cu chemical displacement technique (CDT) is used to form the bottom electrode of ReRAM devices. Compared with conventional deposition methods, the Cu-CDT method has numerous advantages for ReRAM fabrication, including low cost, low temperature fabrication, and the provision of unconsolidated Cu film and large surface roughness. Moreover, the Cu-CDT method is a favorable candidate for overcoming the Cu etching problem and is thus suitable for fabricating ReRAM devices. Using this technique, the surface morphology of a thin Cu film can be easily controlled. The obtained results show that the electric fields during the Forming and SET operations decreased, and the on-state current increased in the RESET operation, as the Cu-CDT displacement time was increased. The Cu-CDT samples exhibited a low operation field, large memory window (>10^6^), and excellent endurance switching cycle characteristics. Moreover, this paper proposes a model to explain the electrical characteristics of ReRAM, which are dependent on the surface morphology.

## 1. Introduction

At present, floating-gate flash memory is the most widely used nonvolatile memory (NVM) [1]. However, flash memory suffers from physical limitations in its fabrication process and reliability problems [2,3]. Therefore, next-generation memories such as magnetoresistive random access memory (MRAM) [4], ferroelectric random access memory (FeRAM) [5], phase–change random access memory (PCRAM) [6], and resistive random access memory (ReRAM) [7,8], are being widely developed to replace flash memory devices. Among these memory types, ReRAM is the most promising NVM [9]. Recently, ReRAM devices have attracted considerable attention because of their advantages such as low power consumption, high operating speed, and favorable scalability [10]. Compared with flash memory devices, ReRAM devices have a simple structure, typically a metal–insulator–metal structure, which results in considerably lower fabrication cost.

Two switching mechanisms of ReRAM devices have been proposed: valence change memory (VCM) and electrochemical metallization (ECM) [11,12]. For VCM-type ReRAM, oxygen vacancies in the insulator are the dominant conduction path and the metal electrode used is usually an inert metal, such as tungsten or platinum [13]. For ECM-type ReRAM, an active metal, such as copper or silver, is used for anode oxidation, and cathode reduction reaction occurs to establish the conduction filament in the switching layer [14]. Cu is more often used in ECM-type ReRAM because it is cheaper than Ag [15]. However, Cu is difficult to dry-etch, and its residue after the etching process contaminates the Si device. This Cu etching problem hinders the development of Cu-based ReRAM.

In this paper, the Cu chemical displacement technique (CDT) is proposed to displace Si into Cu as the metal electrode of a ReRAM device. The Cu-CDT technique is a self-alignment process and can thus prevent the etching problem [16]. Moreover, this technique has numerous advantages such as favorable step coverage, low cost, a low temperature requirement, loose structure provision, and morphology control, which are useful in the development of Cu-based ReRAM devices [17]. To demonstrate the electrical properties of Cu-based ReRAM, an Al/ZnO/Cu-CDT structure was formed. The surface morphology of Cu was also investigated for different Cu-CDT process times. In addition, the operation electric field, memory window, data retention, and endurance of the ReRAM devices using the proposed technique were investigated in this paper.

## 2. Materials and Methods 

**Figure 1:**
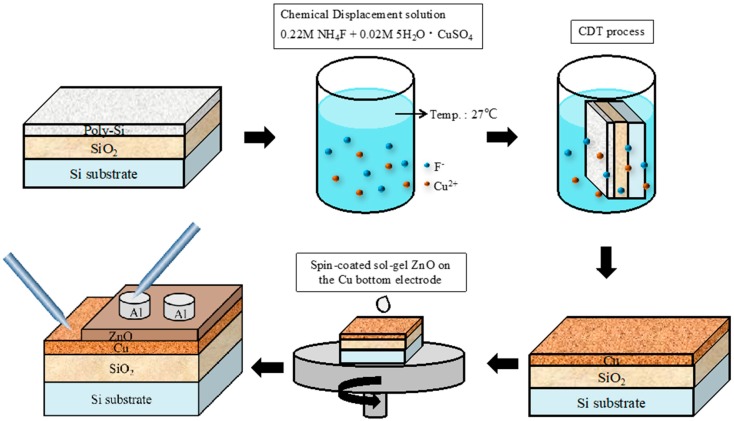
Process flow diagram of fabrication of Cu-CDT ReRAM devices.

Figure 1 illustrates the fabrication process of the Al/ZnO/Cu ReRAM device. After RCA cleaning, a 500 nm TEOS-SiO_2_ buffer oxide and 200 nm poly-Si displacement layer were deposited on a Si substrate through low-pressure chemical vapor deposition. Then, the solution employed in Cu-CDT was mixed with 0.02 M CuSO_4_ and 0.22 M NH_4_F in 100 mL of deionized water. Additionally, 0.05 M ZnO sol-gel solution was fabricated by placing Zn(CH_3_COO)_2_·2H_2_O powder in ethanol (99.5% in purity) and stirring at 60 °C for 2 h. The reactive equation was

$${\mathrm{Zn}(\mathrm{CH}}_{3}{\mathrm{COO})}_{2}\cdot H_{2}O\;\to {\mathrm{Zn}(\mathrm{CH}}_{3}{\mathrm{COO})}_{2}\;+H_{2}O\;↑$$

$${4\mathrm{Zn}(\mathrm{CH}}_{3}{\mathrm{COO})}_{2}\;+\;{2H}_{2}O\to {\mathrm{Zn}}_{4}{O(\mathrm{CH}}_{3}{\mathrm{COO})}_{6}+{2\mathrm{CH}}_{3}\mathrm{COOH}\;↑$$

$${\mathrm{Zn}}_{4}{O(\mathrm{CH}}_{3}{\mathrm{COO})}_{6}\to {\;4\mathrm{ZnO}\;+\;3\mathrm{CH}}_{3}{\mathrm{COOH}\;+\;3\mathrm{CO}}_{2}\;↑$$

The samples were then soaked in the solution at room temperature. To investigate the effect of Cu-CDT, the soaking time was set as 60, 75, and 90 s. In the Cu-CDT technique, fluoride ions (F^−^) attacked poly-Si and released electrons (e^−^). The Cu ions (Cu^2+^) in the solution then accepted the released electrons and reduced to Cu atoms, thereby forming the substrate. The redox reaction was as follows:

$$\begin{array}{l} \begin{array}{ll} \mathrm{half}\;\mathrm{equations}: & (a)\;\mathrm{Si}\;+\;6F^{-}\to {\mathrm{SiF}}_{6}^{2-}+4e^{-}\\ & (b)\;{\mathrm{Cu}}^{2+}+2e^{-}\to \mathrm{Cu} \end{array}\\ \mathrm{total}\;\mathrm{reaction}\;\mathrm{equation}:\;(c)\;\mathrm{Si}+6F^{-}+2{\mathrm{Cu}}^{2+}\to {\mathrm{SiF}}_{6}^{2-}+2\mathrm{Cu} \end{array}$$

A thin Cu film was deposited on the poly-Si substrate after the Cu-CDT process. The samples were then cleaned in deionized water, blow-dried using a N_2_ gun, and baked on a hot plate at 100 °C for 10 min to remove moisture. Thereafter, a 20 nm ZnO thin film was spin-coated using the sol-gel process. The ZnO thin film was formed on the Cu film and used as the switching layer in the ReRAM device. Finally, a 100 nm Al film was deposited using sputtering and a metal mask to define the top electrode of the ReRAM. To demonstrate the device’s switching characteristics, a conventional Cu electrode, deposited using a thermal evaporator, was prepared as the control sample. The switching properties, distributions of SET/RESET voltage, data retention, and endurance characteristics were measured by using the Keithley 4200 system (Tektronix, Beaverton, OR, USA). Surface morphology of the sample is observed by atomic force microscopy (AFM, SOLVER P47-PRO, NT-MDT, Zelenograd, Moscow, Russia) and scanning electron microscopy (SEM, JOEL JSM 6500, JOEL, Tokyo, Japan).

## 3. Results and Discussion

**Figure 2:**
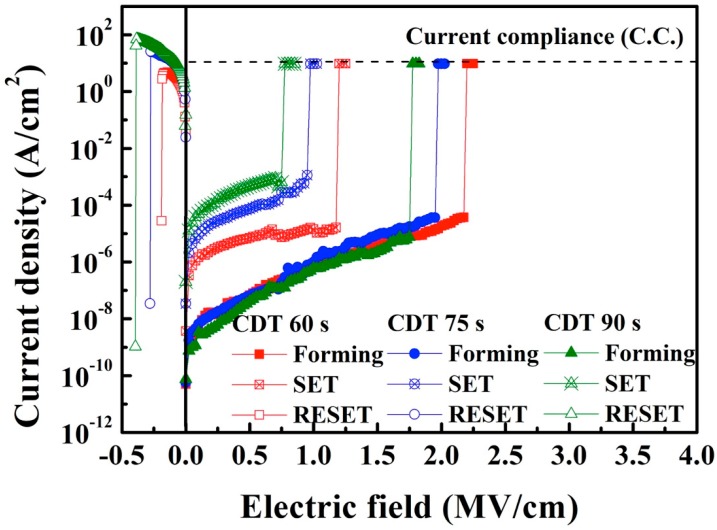
*J–E* characteristic curves of Cu-CDT ReRAM devices.

Figure 2 shows the current density to electric field (*J–E*) switching properties of the Al/ZnO/Cu-CDT ReRAM devices. Direct current (DC) sweep voltage was used for ReRAM switching. Initially, a “Forming” process, in which the devices were operated for the first time, was employed by applying a positive voltage at the Cu electrode. A Cu filament was then established in the resistance switching layer, and the initial high resistance state (HRS) was switched to the low resistance state (LRS). Next, the ReRAM devices were cyclically turned to the HRS (RESET operation) and LRS (SET operation) by applying negative and positive electric fields, respectively. When the Forming and SET operations were in progress, the current was limited to 3 mA to prevent the devices from experiencing hard breakdown.

**Figure 3:**
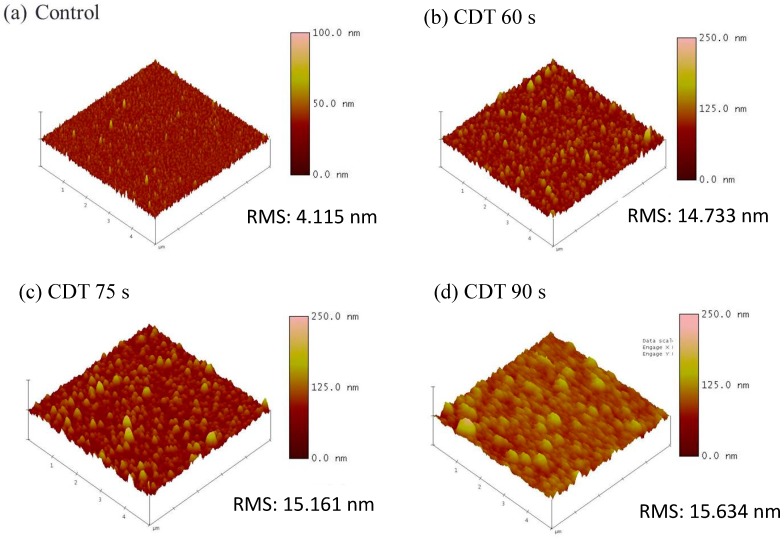
Atomic force microscopy (AFM) images of Cu surface morphology of the (**a**) control; (**b**) CDT 60 s; (**c**) CDT 75 s; and (**d**) CDT 90 s samples.

Figure 2 shows that as the Cu-CDT displacement time was increased, the electric fields during the Forming and SET operations decreased. By contrast, when the ReRAM device was in the RESET operation, the electric field and on-state current increased as the displacement time was increased. To explain this result, AFM was used to observe the Cu film surface. Figure 3 shows the AFM image of the Cu electrode for different displacement times. Figure 3a presents an AFM image of the control sample that deposited the Cu film by using conventional thermal evaporation, and Figure 3b–d are samples deposited using the Cu-CDT method at displacement times of 60, 75, and 90 s, respectively. The surface roughness of the control, CDT 60 s, CDT 75 s, and CDT 90 s samples was 4.115, 14.733, 15.161, and 15.634 nm, respectively. The AFM results show that the surface roughness of the Cu electrode deposited using the Cu-CDT method increased with increasing displacement time. An unconsolidated and rough Cu film could enhance the diffusion of Cu ions into the resistive layer, and thus the filament formed more easily.

**Figure 4:**
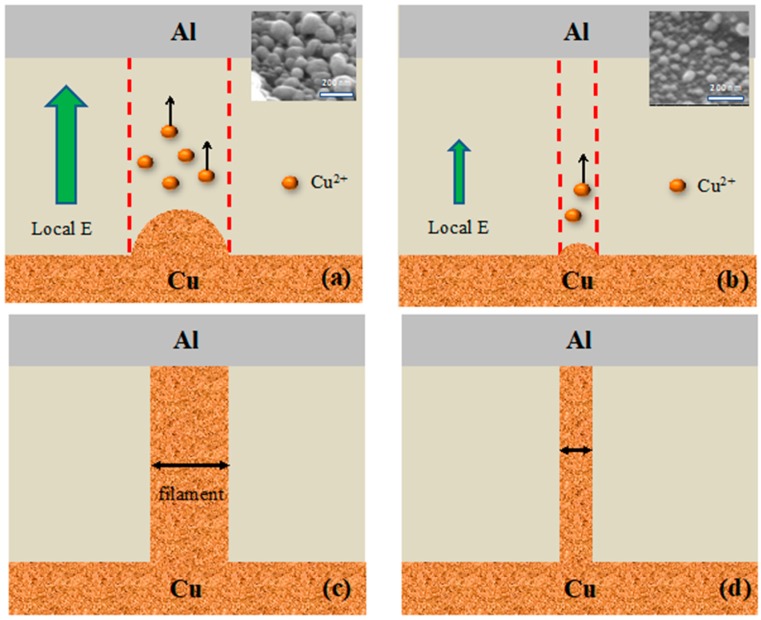
Schematic model of filament formation during the Forming operation for the samples obtained using a (**a**) long (CDT 90 s) and (**b**) short (CDT 60 s) displacement time, and the formed Cu filament after Forming operation of the (**c**) CDT 90 s and (**d**) CDT 60 s sample. Inset: SEM images of the sample surfaces.

A schematic model is illustrated in Figure 4 to describe the mechanism of the ReRAM fabricated using the Cu-CDT method. Figure 4a,b correspond to the samples obtained using a long (CDT 90 s) and short displacement time (CDT 60 s), respectively, in the Forming operation; the insets present SEM images that show the surface morphology of the samples. The AFM and SEM results demonstrate that the CDT 90 s sample exhibited high surface roughness and large grain size. Therefore, the local electric field of the Cu film in the ReRAM device was higher for the CDT 90 s sample. When the Forming operation was in progress, the local electric field in the Cu film enhanced the effect of field emission, promoting the release of Cu ions from the Cu film to the switching layer; thus, the Cu filament in the switching layer was formed more easily (Figure 4a). By contrast, the CDT 60 s sample exhibited a small local electric field, and thus, formation of the filament was difficult, as illustrated in Figure 4b. As a result, the diameter of the formed Cu filament for the CDT 90 s sample was larger than that of the CDT 60 s one (Figure 4c,d). The same mechanism also applies to the Cu-CDT ReRAM in the HRS. The model explains why the applied electric field in the Forming operation and HRS decreased as the Cu-CDT displacement time was increased in Figure 2. It should be noted that the device size in this study is large; however, the trend that surface roughness affects memory performance is still tenable even though the device is getting smaller.

**Figure 5:**
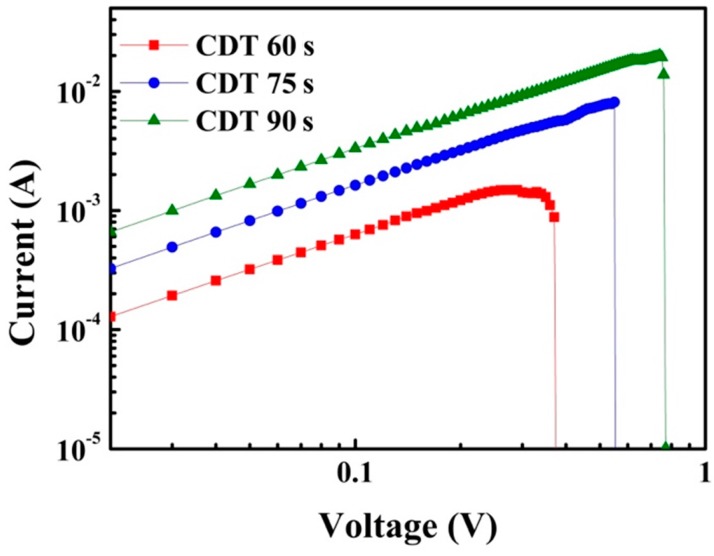
*I–V* curves in logarithmic scale for the ReRAM devices in the LRS.

To prove that the diameter of the Cu filament was dependent on the Cu-CDT displacement time, the *I–V* characteristics were measured when the ReRAM device was in the LRS; that is, when Cu filament existed in the switching layer. Figure 5 shows the *I–V* curves of the ReRAM device in the LRS in double logarithm scale. The samples obtained using a longer Cu-CDT displacement time exhibited a larger current, indicating that Cu filament with large diameter was formed in the device. This was because the Cu grain size increased as the displacement time was increased, and the filament formed according to the Cu size. We have also measured the temperature-dependent resistance variation of the ReRAM device; the results showed that the resistance increases upon increasing the measuring temperature in the LRS (not shown here), which proves that the filament is really composed of metal ions. The same results are also reported in literature [18,19,20].

**Figure 6:**
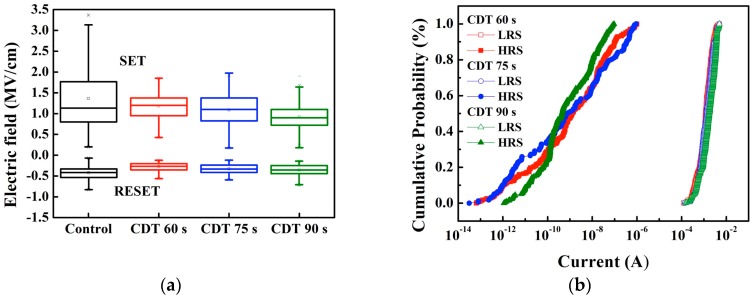
(**a**) Electric field distributions and (**b**) current distributions of the control and Cu-CDT ReRAM devices in the low resistance state (LRS) and high resistance state (HRS).

The distribution of the operation electric field of the Cu-CDT ReRAM devices is shown in Figure 6a. Compared with the control sample, which exhibited large deviation of the electric field during the SET operation, the Cu-CDT sample was more stable and exhibited a lower SET electric field. The Cu film of the control sample was formed by using thermal evaporation; thus, the Cu film was dense and flat compared with the Cu-CDT sample. A dense and flat film is unfavorable for the formation of filament; therefore, the deviation in the electric field increased. By contrast, the large surface roughness of the Cu-CDT sample enhanced the local electric field and caused the Cu ions to move along the direction of the electric field more easily while accelerating the speed of Cu filament formation. Therefore, the Cu filament was established and disrupted at an area where the local electric field was enhanced. Figure 6b shows the current distributions of the Cu-CDT ReRAM devices in the LRS and HRS. Here, the current was obtained using a voltage of 0.1 V to READ the ReRAM devices. It is clearly seen that the devices were more stable in the LRS than in the HRS. When devices were in the LRS, there was no obvious difference between the three samples, whereas the CDT 90 s sample exhibited slightly better performance than the CDT 60 s and 75 s samples when in the HRS.

**Figure 7:**
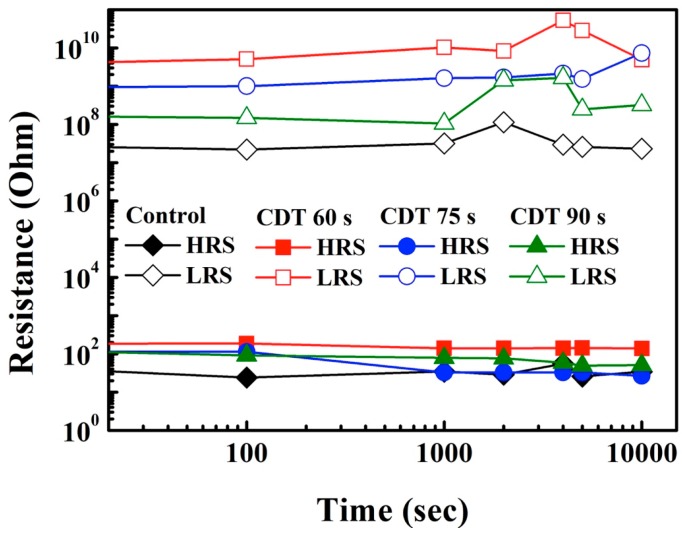
Resistive retention characteristics of the control and Cu-CDT ReRAM devices in the LRS and HRS.

Figure 7 shows the data retention properties of the Al/ZnO/Cu ReRAM devices at room temperature. During the operation, a DC sweep was applied to switch the samples into the LRS/HRS, and a voltage of 0.1 V was applied to READ the ReRAM devices. Because of random vibrations, the Cu ions contacted the Cu filament and transformed into Cu atoms when a READ voltage was applied. This resulted in the devices being more stable in the LRS than in the HRS. Moreover, although all samples could retain data at 10,000 s with only a small memory window, the *R*_ON_/*R*_OFF_ ratios of the Cu-CDT samples were larger than that of the control sample. The *R*_ON_/*R*_OFF_ ratio increased as the Cu-CDT displacement time was decreased. The *R*_ON_/*R*_OFF_ ratios of the control, CDT 60 s, CDT 75 s, and CDT 90 s samples were $7.7\times 10^{4}$, $3.1\times 10^{6}$, $2.8\times 10^{6}$, and $4.6\times 10^{5}$, respectively. These results were obtained because of the diameter of the local electric field caused by the Cu grain size. For the CDT 60 s device, the lower Cu concentration in the switching layer induced a higher resistance in the HRS, and thus, a larger *R*_ON_/*R*_OFF_ ratio was obtained.

**Figure 8:**
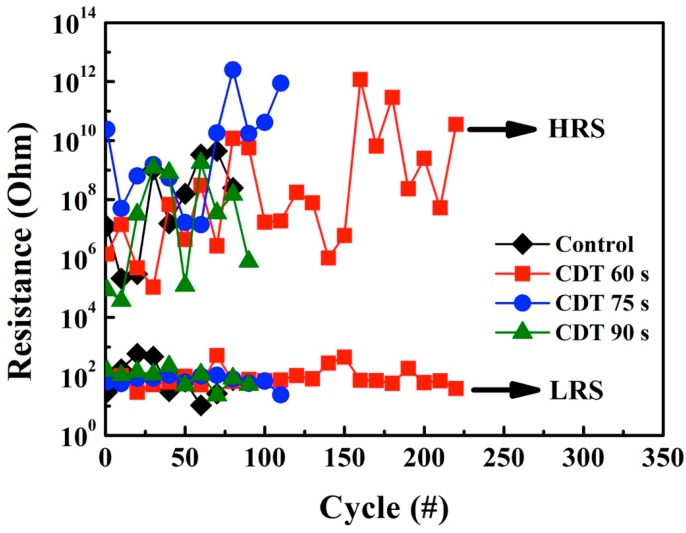
Endurance characteristics of the control and Cu-CDT ReRAM devices in the LRS and HRS.

The endurance characteristics of the ReRAM devices are illustrated in Figure 8. To measure the endurance of the ReRAM devices, a DC sweep cycle was applied and a voltage of 0.1 V was applied to READ. The endurance increased as the displacement time was decreased. The CDT 60 s sample has the optimal endurance property, which can sustain SET/RESET times over 200 cycles. Compared with other studies that used similar material, the CDT 60 s sample exhibits good reliability [21,22]. The failure of ReRAM devices in an endurance cycle is caused by the Joule heating effect. The longer the displacement time used to obtain the sample, which implies a larger diameter of the Cu filament, the more energy is required to disrupt the filament. During the RESET operation, the local electric field decreased, and thus, large-diameter Cu filament formed with more difficulty. By contrast, a smaller diameter of the Cu filament caused a weaker Joule heating effect, which improved the endurance of the ReRAM device.

## 4. Conclusions

The electrical performance of Al/ZnO/Cu ReRAM devices obtained using the Cu-CDT technique has been demonstrated in this paper. We used CDT to replace poly-silicon into copper, which then served as the bottom electrode of the devices. Results demonstrated that electrode surface morphology considerably affects memory performance. The sample with appropriate roughness of the Cu-CDT surface had enhanced local electric field, thereby accelerating the formation of the Cu filament. By contrast, the sample with excessive local roughness of the Cu-CDT surface induced grain growth. Compared with the control sample, the Cu-CDT ReRAM device had lower operation field strength and higher reliability. Furthermore, the RESET voltage of the Cu-CDT ReRAM device decreased when the Cu-CDT process time was decreased. The device exhibited large memory windows (*R*_ON_/*R*_OFF_ > 10^6^) and excellent endurance during switching cycles when the CDT 60 s process was employed. Thus, Cu-CDT is a favorable technique for overcoming the etching problem, is compatible with current IC technology, and can be employed to fabricate high-performance memory devices.
